# Cytokine and interleukin profile in patients with headache and COVID-19: A pilot, CASE-control, study on 104 patients

**DOI:** 10.1186/s10194-021-01268-w

**Published:** 2021-06-04

**Authors:** Javier Trigo, David García-Azorín, Álvaro Sierra-Mencía, Álvaro Tamayo-Velasco, Pedro Martínez-Paz, Eduardo Tamayo, Angel Luis Guerrero, Hugo Gonzalo-Benito

**Affiliations:** 1 Headache Unit, Neurology Service, University Clinical Hospital of Valladolid, 3 Ramón y Cajal Ave, 47003 Valladolid, Spain; 2 Haematology and Haemotherapy Service, University Clinical Hospital of Valladolid, 3 Ramón y Cajal Ave, 47003 Valladolid, Spain; 3 Department of Surgery, Faculty of Medicine, University of Valladolid, 7 Ramón y Cajal Ave, 47005 Valladolid, Spain; 4 Anaesthesiology and Resuscitation Service, University Clinical Hospital of Valladolid, 3 Ramón y Cajal Ave, 47003 Valladolid, Spain; 5 Department of Medicine, Dermatology and Toxicology, Faculty of Medicine, University of Valladolid, 7 Ramón y Cajal Ave, 47005 Valladolid, Spain; 6 Institute of Health Sciences of Castile and Leon (ICSCYL), Santa Clara Square, 42002 Soria, Spain

**Keywords:** COVID-19, Headache disorders, Interleukins, Cytokines, Immune system

## Abstract

**Background:**

The presence of headache during the acute phase of COVID-19 could be associated with the innate response and the cytokine release. We aim to compare the cytokine and interleukin profile in hospitalized COVID-19 patients at the moment of admission with and without headache during the course of the disease.

**Methods:**

An observational analytic study with a case control design was performed. Hospitalized patients from a tertiary hospital with confirmed COVID-19 disease were included. Patients were classified into the headache or the control group depending on whether they presented headache not better accounted for by another headache disorder other than acute headache attributed to systemic viral infection. Several demographic and clinical variables were studies in both groups. We determined the plasmatic levels of 45 different cytokines and interleukins from the first hospitalization plasma extraction in both groups.

**Results:**

One hundred and four patients were included in the study, aged 67.4 (12.8), 43.3% female. Among them, 29 (27.9%) had headache. Patients with headache were younger (61.8 vs. 69.5 years, *p* = 0.005) and had higher frequency of fever (96.6 vs. 78.7%, *p* = 0.036) and anosmia (48.3% vs. 22.7%, *p* = 0.016). In the comparison of the crude median values of cytokines, many cytokines were different between both groups. In the comparison of the central and dispersion parameters between the two groups, GROa, IL-10, IL1RA, IL-21, IL-22 remained statistically significant. After adjusting the values for age, sex, baseline situation and COVID-19 severity, IL-10 remained statistically significant (3.3 vs. 2.2 ng/dL, *p* = 0.042), with a trend towards significance in IL-23 (11.9 vs. 8.6 ng/dL, *p* = 0.082) and PIGF1 (1621.8 vs. 110.6 ng/dL, *p* = 0.071).

**Conclusions:**

The higher levels of IL-10 -an anti-inflammatory cytokine- found in our sample in patients with headache may be explained as a counteract of cytokine release, reflecting a more intense immune response in these patients.

**Supplementary Information:**

The online version contains supplementary material available at 10.1186/s10194-021-01268-w.

## Background

Headache is one of the most frequent symptoms of Coronavirus disease 2019 (COVID-19) [1]. In most cases it occurs within the first days after the onset of symptoms [2]. It is typically described as bilateral, frontal, pressing in quality and of severe intensity [3]. The profile of patients who present headache over the course of COVID-19 disease is unique, being associated with: i) demographic variables, as female sex, younger age, and prior history of headache [4] ii) a higher frequency of some clinical variables, as anosmia [5], myalgia, fever [4] and iii) a different laboratory profile, including a higher C-reactive protein, Interleukine-6 and D-dimer and a lower lymphocyte count [6].

The early onset [2], the unspecific phenotype [7], the similarities with headache associated with other systemic viral infections [8], and the coexistence with arthralgia, cough, lightheadedness and mialya [4] made some authors to suggest that it might be associated with the innate immune response and the cytokine release [4]. Cytokine storm has been described during COVID-19 infection, and prior studies have observed that patients with higher levels of selected cytokines and interleukins presented a more severe clinical presentation [9, 10]. Bearing in mind that headache is within the most common symptoms of COVID-19 (14,1%) [1], it seems plausible the presence of a specific headache and cytokine profile. However, this hypothesis has not been addressed yet. We present a pilot study in order to evaluate the cytokine and interleukin profile of COVID-19 patients with headache, compared with COVID-19 patients without headache. If this hypothesis is confirmed, new knowledge would support the molecular mechanisms underlying the headache related to COVID-19. These results could serve both to improve COVID-19 management and to describe new therapeutic targets that would have a direct impact on the quality of life of patients.

## Methods

### Design

This is an observational analytic study with a case-control design. The study population included patients with COVID-19 divided in two groups, depending on the presence or absence of headache. The study period was from March 8th to April 11th, 2020. The eligibility criteria have been extensively described in other studies [2, 4–7], and briefly included patients with confirmed COVID-19 disease that were hospitalized. The infection was confirmed by real-time polymerase chain reaction test and/or serum antibody test. We excluded patients not admitted from the emergency department or those patients with unavailable electronic health records. The recruitment strategy was carried out by randomly enrolling one out of five patients from patients consecutively admitted to the hospital, since the first case diagnosed in our hospital Patients were classified into the headache or the control group depending on whether they presented headache not better accounted for by another headache disorder other than acute headache attributed to systemic viral infection [11]. The headache was evaluated by a neurologist with expertise on headache disorders. The study was done in the University Clinical Hospital of Valladolid, Spain. The Ethics Review Board approved the study (PI-20-1751, PI-20-1717).

### Study objectives

The primary objective of the study was to describe the specific cytokine and interleukin profile of patients with headache during COVID-19.

### Variables

We studied several demographic and clinical variables [2, 4–7] (supplementary materials). We analysed the clinical outcome, including the COVID-19 severity [12] (full definition in supplementary materials), and the need of intensive care unit (ICU) admission, ventilatory support, oxygen therapy and all-cause in-hospital mortality. As experimental parameters, we analysed the cytokine profile as summarized below.

### Cytokine profile

The plasma samples were obtained from the antecubital vein, collected at the first extraction that was done in each patient during the hospitalization period. We use the 45-plex Human XL Cytokine Luminex Performance Panel (R&D) kit (Invitrogen, Thermofisher Scientific) following the manufacturer’s recommendations and using a Luminex™ MAGPIX™ Instrument System. In summary, it is a high resolution and sensitivity immunoassay based on enzyme-linked immunosorbent assay (ELISA) method. With that technology, the concentration of the following cytokines was quantified: brain-derived neurotrophic factor (BDNF), Eotaxin/CCL11, epidermal growth factor (EGF), fibroblast growth factor 2 (FGF-2), granulocyte macrophage colony-stimulating factor (GM-CSF), growth-regulated oncogene (GRO) alpha/chemokine (C-X-C motif) ligand 1(CXCL1), hepatocyte growth factor (HGF), nerve growth factor (NGF) beta, leukaemia inhibitory factor (LIF), interferon (IFN) alpha, IFN gamma, interleukin (IL)-1 alpha, IL-1 beta, interleukin 1 receptor antagonist (IL-1RA), IL-2, IL-4, IL-5, IL-6, IL-7, IL-8/CXCL8, IL-9, IL-10, IL-12 p70, IL-13, IL-15, IL-17A, IL-18, IL-21, IL-22, IL-23, IL-27, IL-31, interferon gamma-induced protein 10 (IP-10)/ chemokine (C-X-C motif) ligand 10 (CXCL10), monocyte chemoattractant protein 1 (MCP-1)/chemokine (C-C motif) ligand 2 (CCL2), macrophage inflammatory protein 1 (MIP-1) alpha/ (chemokine (C-C-motif) ligand 3(CCL3), regulated upon activation normal T Cell expressed and presumably secreted (RANTES)/ chemokine (C-C motif) ligand 5 (CCL5), stromal-cell derived factor 1 (SDF-1) alpha/CXCL12, tumoral necrosis factor (TNF) alpha, TNF beta/lymphotoxin alpha (LTA), platelet-derived growth factor (PDGF)-BB, placental growth factor (PIGF-1), stem cell factor (SCF), vascular endothelial growth factor (VEGF)-A and VEGF-D.

### Statistical analysis

We present qualitative variables as frequency and percentage, and quantitative variables as mean and standard deviation (SD) if the distribution was normal, or as median and inter-quartile range (IQR) if not. For hypothesis testing, we used Fisher’s Exact test, Student’s t-test or Mann-Whitney U test.

We firstly analysed and compared baseline clinical and demographic variables in both groups (patients with headache and patients without headache).

Since this was a pilot study, the sample size was relatively small and there were many different analysed variables, we tried to validate the analysis by testing the analysis in terms of central tendency, dispersion and after adjusting for potential confounders. First, in order to compare the medians of the samples, since we expected a small sample with few outliers, we used Mood’s median test to compare the medians of headache patients and non-headache patients. Second, we tested the differences in shape and spread of the values by using the Mann-Whitney U tests. Third, we compared the parameters adjusting for covariates by using ANCOVA. The adjusted variables included age, sex, days since the onset of COVID-19 and severity of the disease.

We considered results as statistically significant if the *p*-value was < 0.05, however, given that this was a pilot study, we aimed to report also those variables with a trend towards signification (*p* < 0.1), since researchers might prioritize their study in future research projects. We did not estimate sample size in advance. Statistical analysis was performed with SPSS v26 (IBM Corp. Armonk, NY).

## Results

One hundred and four patients were included in the study, aged 67.4 (12.8), 43.3% female. Among them, 29 (27.9%) had headache. Patients with headache were younger (61.8 vs. 69.5 years, *p* = 0.005) and had higher frequency of fever (96.6 vs. 78.7%, *p* = 0.036) and anosmia (48.3% vs. 22.7%, *p* = 0.016). We did not observe differences in other demographic variables, frequency of prior history conditions, clinical symptoms or variables related with the clinical outcome (Table 1).

**Table 1:** Demographic, clinical and outcome variables

Variable	Entire study sample (*n* = 104)	Headache patients (*n* = 29)	Non-headache patients (*n* = 75)	*p*-value
Mean age (years)	67.4 (12.8)	61.8 (13.6)	69.5 (11.9)	0.005
Female sex (n, %)	45 (43.3%)	15 (51.7%)	30 (40.0%)	0.378
Median Rankin scale	0 [0–0]	0 [0–0]	0 [0–1]	0.409
Prior history of hypertension (n, %)	52 (50.0%)	11 (37.9%)	41 (54.7%)	0.189
Prior history of diabetes (n, %)	20 (19.2%)	6 (20.7%)	14 (18.7%)	0.788
Prior history of smoking (n, %)	24 (23.1%)	5 (17.2%)	19 (25.3%)	0.446
Prior history of cardiac disorders (n, %)	28 (26.9%)	7 (24.1%)	21 (28.0%)	0.808
Prior history of pulmonary disorders (n, %)	25 (24.0%)	7 (24.1%)	18 (24.0%)	> 0.999
Prior history of cancer (n, %)	10 (9.6%)	4 (13.8%)	6 (8.0%)	0.460
Prior history of immunosuppression (n, %)	3 (2.9%)	1 (3.4%)	2 (2.7%)	> 0.999
Prior history of neurological disorders (n, %)	15 (14.4%)	4 (13.8%)	11 (14.7%)	> 0.999
Prior history of headache (n, %)	4 (3.8%)	1 (3.4%)	3 (4.0%)	> 0.999
Time between the onset of symptoms and the ER visit (days)	7.2 (4.9)	7.5 (3.4)	7.0 (5.4)	0.683
Presence of arthralgia (n, %)	6 (5.8%)	3 (10.3%)	3 (4.0%)	0.345
Presence of asthenia (n, %)	45 (43.3%)	17 (58.6%)	28 (37.3%)	0.077
Presence of weakness (n, %)	19 (18.3%)	7 (24.1%)	12 (16.0%)	0.398
Presence of diarrhoea (n, %)	41 (39.4%)	9 (31.0%)	32 (42.7%)	0.371
Presence of dyspnoea (n, %)	55 (52.9%)	15 (51.7%)	40 (53.3%)	> 0.999
Presence of chest pain (n, %)	20 (19.2%)	5 (17.2%)	15 (20.0%)	> 0.999
Presence of expectoration (n, %)	11 (10.6%)	1 (3.4%)	10 (13.3%)	0.284
Presence of fever (n, %)	87 (83.7%)	28 (96.6%)	59 (78.7%)	0.036
Presence of anosmia (n, %)	31 (29.8%)	14 (48.3%)	17 (22.7%)	0.016
Presence of light-headedness (n, %)	10 (9.6%)	4 (13.8%)	6 (8.0%)	0.460
Presence of myalgia (n, %)	27 (26.0%)	10 (34.5%)	17 (22.7%)	0.224
Presence of odynophagia (n, %)	9 (8.7%)	2 (6.9%)	7 (9.3%)	> 0.999
Presence of cough (n, %)	81 (77.9%)	24 (82.8%)	57 (76.0%)	0.601
Presence of vomiting (n, %)	6 (5.8%)	2 (6.9%)	4 (5.3%)	0.670
Mild disease (n, %)	3 (2.9%)	2 (6.9%)	1 (1.3%)	0.187
Pneumonia (n, %)	17 (16.3%)	5 (17.2%)	12 (16.0%)	> 0.999
Severe pneumonia (n, %)	45 (43.3%)	11 (37.9%)	34 (45.3%)	0.518
ARDS (n, %)	38 (36.5%)	11 (37.9%)	27 (36.0%)	> 0.999
ICU admission (n, %)	31 (29.8%)	8 (27.6%)	23 (30.7%)	0.815
Ventilatory support (n, %)	31 (29.8%)	8 (27.6%)	23 (30.7%)	0.815
Need of oxygen therapy (n, %)	83 (79.8%)	21 (72.4%)	62 (82.7%)	0.280
Death (n, %)	20 (19.2%)	3 (10.3%)	17 (22.7%)	0.178

Regarding the central tendency measures, in the comparison of the crude median values of cytokines, we observed that patients with headache had higher median values of GROa, IFN-gamma, IL-10, IL-13, IL-15, IL-17a, IL-21, IL-22, IL-27 and IL-6 (Table 2).

**Table 2:** comparison of the crude median values of cytokines

Variable	Entire study sample (*n* = 104)	Headache patients (*n* = 29)	Non-headache patients (*n* = 75)	*P* value
BDNF	56.5 (35.1–160.9)	54.3 (35. 4-154)	60.5 (33. 9-184.5)	0.512
EGF	2.1 (0. 7-8.3)	2.7 (1. 2-11.2)	1.7 (0. 5-7.3)	0.126
Eotaxin	13.8 (9. 6-19.2)	12.8 (9. 1-20.6)	13.9 (10. 3-19.1)	0.512
FGF2	0.9 (0. 3-2.7)	1.5 (0. 6-3.0)	0.6 (0. 2-2.2)	0.274
GMCSF	11.3 (5. 4-29.7)	13.3 (7. 9-40.7)	10.5 (4. 3-28.1)	0.126
GROa	3.0 (1. 3-5.6)	3.8 (2. 4-7.1)	2.7 (1. 2-4.5)	0.049
HGF	162 (104. 4-322)	167 (87. 2-281.5)	161 (104. 9-384-5)	0.512
IFNa	0.5 (0. 1-1.8)	0.5 (0. 2-1.2)	0.5 (0. 1-2.1)	0.827
IFNg	8.8 (5. 6-12.3)	10.4 (7. 5-15.1)	7.9 (5. 2-11.7)	0.016
IL1a	2.3 (0. 4-8.5)	3.0 (0. 7-13.9)	2.2 (0. 4-8.1)	0.827
IL1b	6.3 (2. 7-13.1)	7.6 (4. 1-15.0)	5.5 (2. 6-13.8)	0.126
IL10	1.7 (1. 1-3.8)	2.5 (1. 6-4.3)	1.4 (1.0–3.3)	0.004
IL12p70	3.5 (2. 3-5.3)	4.3 (3.0–5.6)	3.1 (2. 1-5)	0.126
IL13	1.9 (0. 9-3.7)	2.7 (1. 5-4.7)	1.7 (0. 8-3.3)	0.016
IL15	13.2 (6. 6-24.5)	16 (10.0–24.3)	12.6 (5. 8-26.4)	0.049
IL17a	7.0 (3. 1-18.7)	11.8 (4. 5-19.6)	5.3 (2. 7-17.6)	0.049
IL18	46.4 (24. 2-77.8)	25.6 (29. 6-84.5)	49 (23. 5-75.9)	0.512
IL1RA	579 (230. 6-1293.7)	986.5 (409. 6-1895)	466 (165–981)	0.126
IL2	13.0 (6. 9-26.6)	13.3 (7. 5-30.7)	12.6 (6. 6-26.2)	0.827
IL21	3.3 (0. 6-11.5)	7.5 (2. 6-16.7)	1.8 (0. 5-8.8)	0.016
IL22	3.6 (0. 3-22.8)	12.9 (1. 8-57.6)	2.1 (0. 2-15.2)	0.016
IL23	7.6 (3. 8-12.4)	10.0 (6. 3-13.5)	6.8 (3. 4-11.5)	0.274
IL27	15.8 (4. 9-38.8)	28.1 (10. 7-90.1)	12.7 (4. 5-32.6)	0.004
IL31	5.2 (2. 3-9.7)	7.2 (4. 3-10.7)	3.9 (2. 1-9.3)	0.126
IL4	5.4 (2. 8-9.7)	7.8 (4. 1-10.0)	5.0 (2. 6-9.0)	0.126
IL5	4.9 (1. 3-18.5)	7.6 (3.0–25.2)	5.0 (2. 6-8.9)	0.126
IL6	12.6 (5. 6-29.7)	19.6 (7. 2-35.1)	10.0 (4. 6-24.3)	0.049
IL7	1.6 (0. 6-3.6)	1.7 (0. 8-3.4)	1.4 (0. 6-4.1)	0.827
IL8	1.9 (0. 6-4.3)	1.8 (1. 1-2.8)	2.0 (0. 5-5.4)	0.827
IL9	2.1 (1.0–4.2)	2.9 (1. 7-4.9)	1.7 (0. 9-3.9)	0.126
IP1b	48.6 (32. 5-77.6)	51.2 (33. 6-90.4)	48 (29. 7-71.1)	0.827
IP10	46.2 (29. 7-71.5)	49.8 (35. 4-68.9)	45.3 (27. 4-71.7)	0.126
LIF	14.9 (7. 8-25.1)	17.5 (10. 1-25.6)	14.6 (6. 8-25.3)	0.437
MCP1	38.1 (24. 1-56.2)	34.3 (25. 3-48.8)	42.0 (23. 8-57.1)	0.126
MIP1a	3.2 (1. 4-13.1)	3.9 (2. 2-11.0)	3.1 (1. 3-13.9)	0.512
NGFb	4.1 (2. 7-5.9)	4.6 (3. 5-6.0)	3.7 (2. 5-5.7)	0.274
PDGFBB	303.4 (89. 7-771)	286 (77. 6-777.3)	331.3 (106–782)	0.827
PIGF1	5.0 (0. 7-65.0)	4.4 (0. 9-72.3)	5.4 (0. 6-60.1)	0.827
RANTES	22.5 (16. 8-36.3)	19.6 (15. 4-31.3)	24.2 (17. 8-36.7)	0.126
SCF	6.5 (3. 4-10.9)	6.9 (2. 3-10.1)	6.2 (3. 5-11.9)	0.274
SDF1a	677.7 (485–1094.8)	786 (476–1735)	628.5 (48 4-1019)	0.126
TNFa	5.9 (3. 4-14.3)	8.0 (4.4–14.8)	5.7 (3. 2-12.9)	0.274
TNFb	3.2 (1. 7-5.9)	4.2 (2. 6-6.5)	2.6 (1. 5-5.4)	0.126
VEGFA	124.1 (69.0–286.4)	124.5 (85. 4-333)	123.8 (64. 6-251)	0.827
VEGFD	12.6 (7. 2-20.6)	12.6 (5. 4-19.7)	12.6 (7. 4-20.6)	0.827

**Fig. 1:**
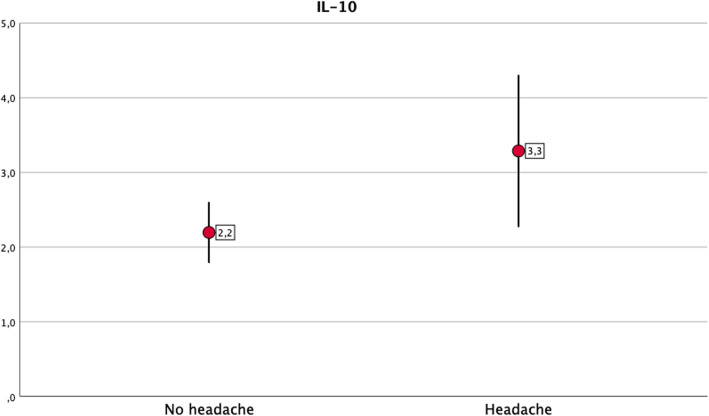
Comparison of median IL-10 levels between COVID-19 patients with and without headache by ANCOVA test, adjusting for age, sex, baseline disability and COVID-19 severity

**Fig. 2:**
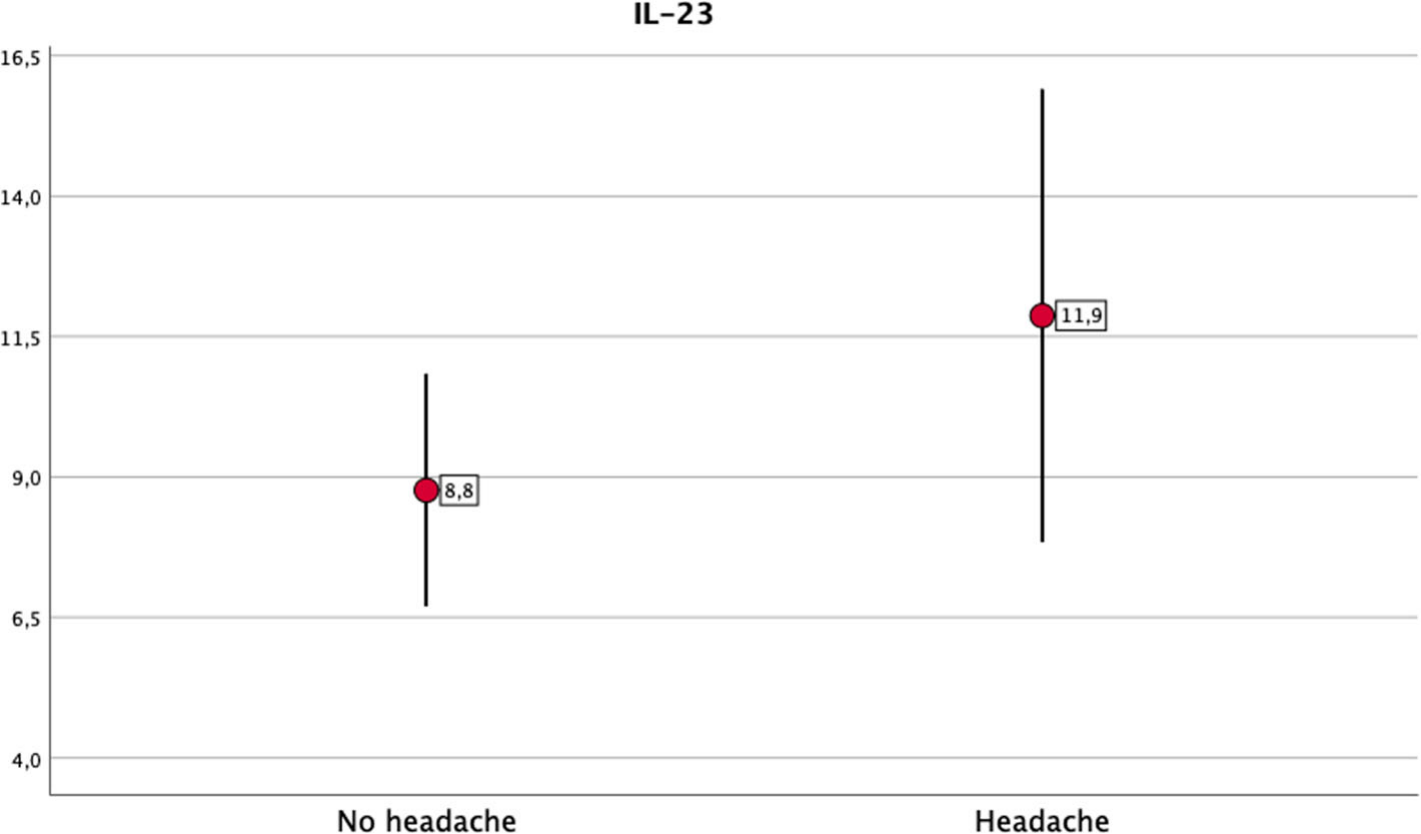
Comparison of median IL-23 levels between COVID-19 patients with and without headache by ANCOVA test, adjusting for age, sex, baseline disability and COVID-19 severity

**Fig. 3:**
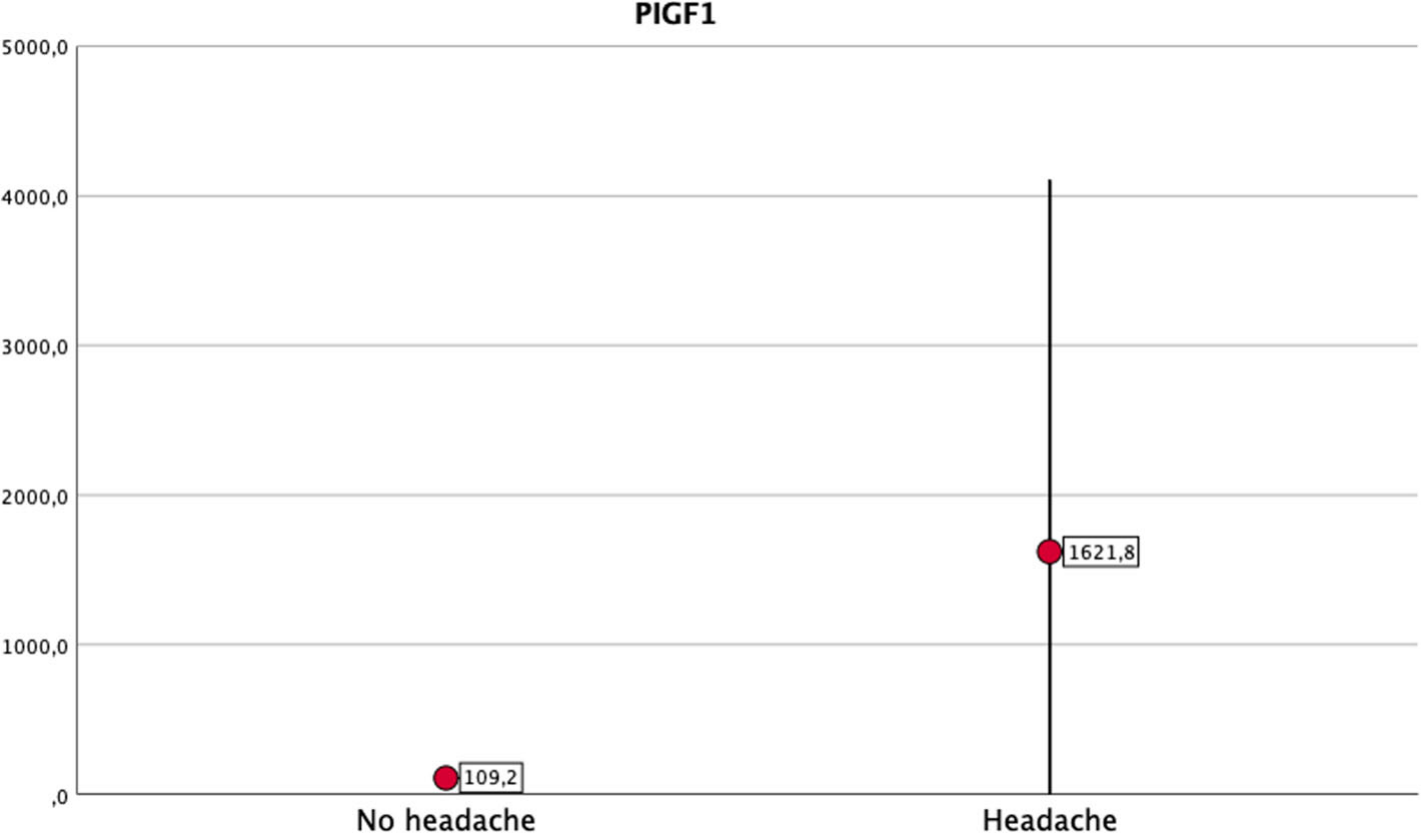
Comparison of median PIGF1 levels between COVID-19 patients with and without headache by ANCOVA test, adjusting for age, sex, baseline disability and COVID-19 severity

In regard to the central and dispersion of the parameters, in the comparison of the shape and spread between the two groups, GROa, IL-10, IL1RA, IL-21, IL-22 remained statistically significant, while there were trends towards signification (*p* < 0.1) in FGF-2, IFNg, IL12p70, IL-23, IL-27, IL31, IL-6, IL-9 and TNF-b (Table 3). After adjusting the values for age, sex, baseline situation and COVID-19 severity, only IL-10 remained statistically significant (3.3 vs. 2.2 ng/dL, *p* = 0.042) with a trend towards signification in IL-23 (11.9 vs. 8.6 ng/dL, *p* = 0.082) and PIGF1 (1621.8 vs. 110.6 ng/dL, *p* = 0.071) (Figs. 1, 2 and 3) (Table 4).

**Table 3:** Comparison of cytokines and interleukins by MWU test

Variable	Entire study sample	Headache patients	Non-headache patients	*P* value
BDNF	56.5 (35. 1-160.9)	54.3 (35. 4-154)	60.5 (33. 9-184.5)	0.928
EGF	2.1 (0. 7-8.3)	2.7 (1. 2-11.2)	1.7 (0. 5-7.3)	0.191
Eotaxin	13.8 (9. 6-19.2)	12.8 (9. 1-20.6)	13.9 (10. 3-19.1)	0.460
FGF2	0.9 (0. 3-2.7)	1.5 (0. 6-3.0)	0.6 (0. 2-2.2)	0.064
GMCSF	11.3 (5. 4-29.7)	13.3 (7. 9-40.7)	10.5 (4. 3-28.1)	0.085
GROa	3.0 (1. 3-5.6)	3.8 (2. 4-7.1)	2.7 (1. 2-4.5)	0.046
HGF	162 (104. 4-322)	167 (87. 2-281.5)	161 (104. 9-384-5)	0.625
IFNa	0.5 (0. 1-1.8)	0.5 (0. 2-1.2)	0.5 (0. 1-2.1)	0.997
IFNg	8.8 (5. 6-12.3)	10.4 (7. 5-15.1)	7.9 (5. 2-11.7)	0.073
IL1a	2.3 (0. 4-8.5)	3.0 (0. 7-13.9)	2.2 (0. 4-8.1)	0.402
IL1b	6.3 (2. 7-13.1)	7.6 (4. 1-15.0)	5.5 (2. 6-13.8)	0.120
IL10	1.7 (1. 1-3.8)	2.5 (1. 6-4.3)	1.4 (1.0–3.3)	0.008
IL12p70	3.5 (2. 3-5.3)	4.3 (3.0–5.6)	3.1 (2. 1-5)	0.061
IL13	1.9 (0. 9-3.7)	2.7 (1. 5-4.7)	1.7 (0. 8-3.3)	0.101
IL15	13.2 (6. 6-24.5)	16 (10.0–24.3)	12.6 (5. 8-26.4)	0.295
IL17a	7.0 (3. 1-18.7)	11.8 (4. 5-19.6)	5.3 (2. 7-17.6)	0.139
IL18	46.4 (24. 2-77.8)	25.6 (29. 6-84.5)	49 (23. 5-75.9)	0.968
IL1RA	579 (230. 6-1293.7)	986.5 (409. 6-1895)	466 (165–981)	0.03
IL2	13.0 (6. 9-26.6)	13.3 (7. 5-30.7)	12.6 (6. 6-26.2)	0.550
IL21	3.3 (0. 6-11.5)	7.5 (2. 6-16.7)	1.8 (0. 5-8.8)	0.022
IL22	3.6 (0. 3-22.8)	12.9 (1. 8-57.6)	2.1 (0. 2-15.2)	0.027
IL23	7.6 (3. 8-12.4)	10.0 (6. 3-13.5)	6.8 (3. 4-11.5)	0.065
IL27	15.8 (4. 9-38.8)	28.1 (10. 7-90.1)	12.7 (4. 5-32.6)	0.067
IL31	5.2 (2. 3-9.7)	7.2 (4. 3-10.7)	3.9 (2. 1-9.3)	0.068
IL4	5.4 (2. 8-9.7)	7.8 (4. 1-10.0)	5.0 (2. 6-9.0)	0.135
IL5	4.9 (1. 3-18.5)	7.6 (3.0–25.2)	5.0 (2. 6-8.9)	0.105
IL6	12.6 (5. 6-29.7)	19.6 (7. 2-35.1)	10.0 (4. 6-24.3)	0.099
IL7	1.6 (0. 6-3.6)	1.7 (0. 8-3.4)	1.4 (0. 6-4.1)	0.934
IL8	1.9 (0. 6-4.3)	1.8 (1. 1-2.8)	2.0 (0. 5-5.4)	0.991
IL9	2.1 (1.0–4.2)	2.9 (1. 7-4.9)	1.7 (0. 9-3.9)	0.064
IP1b	48.6 (32. 5-77.6)	51.2 (33. 6-90.4)	48 (29. 7-71.1)	0.295
IP10	46.2 (29. 7-71.5)	49.8 (35. 4-68.9)	45.3 (27. 4-71.7)	0.547
LIF	14.9 (7. 8-25.1)	17.5 (10. 1-25.6)	14.6 (6. 8-25.3)	0.449
MCP1	38.1 (24. 1-56.2)	34.3 (25. 3-48.8)	42.0 (23. 8-57.1)	0.396
MIP1a	3.2 (1. 4-13.1)	3.9 (2. 2-11.0)	3.1 (1. 3-13.9)	0.423
NGFb	4.1 (2. 7-5.9)	4.6 (3. 5-6.0)	3.7 (2. 5-5.7)	0.193
PDGFBB	303.4 (89. 7-771)	286 (77. 6-777.3)	331.3 (106–782)	0.888
PIGF1	5.0 (0. 7-65.0)	4.4 (0. 9-72.3)	5.4 (0. 6-60.1)	0.825
RANTES	22.5 (16. 8-36.3)	19.6 (15. 4-31.3)	24.2 (17. 8-36.7)	0.110
SCF	6.5 (3. 4-10.9)	6.9 (2. 3-10.1)	6.2 (3. 5-11.9)	0.599
SDF1a	677.7 (485–1094.8)	786 (476–1735)	628.5 (48 4-1019)	0.335
TNFa	5.9 (3. 4-14.3)	8.0 (4.4–14.8)	5.7 (3. 2-12.9)	0.333
TNFb	3.2 (1. 7-5.9)	4.2 (2. 6-6.5)	2.6 (1. 5-5.4)	0.055
VEGFA	124.1 (69.0–286.4)	124.5 (85. 4-333)	123.8 (64. 6-251)	0.555
VEGFD	12.6 (7. 2-20.6)	12.6 (5. 4-19.7)	12.6 (7. 4-20.6)	0.432

**Table 4:** Comparison of cytokines and interleukins by ANCOVA test, adjusting for age sex, baseline disability and COVID-19 severity

Variable	Entire study sample (*n* = 104)	Headache patients (*n* = 29)	Non-headache patients (*n* = 75)	*P* value
BDNF	159.9 (230.1)	167.6 (242.6)	156.8 (226.7)	0.912
EGF	8.6 (18.4)	7.6 (10.2)	8.9 (20.7)	0.766
Eotaxin	16.2 (9.4)	15.6 (10.0)	16.5 (9.2)	0.878
FGF2	2.7 (6.6)	3.1 (5.6)	2.5 (6.9)	0.799
GMCSF	25.1 (33.3)	27.4 (29.1)	24.2 (34.9)	0.476
GROa	4.4 (4.5)	4.9 (3.3)	4.2 (4.9)	0.218
HGF	348.5 (622.3)	237.2 (273.3)	392.1 (711.1)	0.374
IFNa	2.36 (6.4)	2.1 (4.5)	2.5 (7.0)	0.952
IFNg	11.4 (9.4)	12.8 (9.7)	10.8 (9.3)	0.246
IL1a	64.2 (287.0)	130.9 (440.1)	38.1 (195.9)	0.238
IL1b	9.7 (10.2)	10.3 (8.0)	9.5 (10.9)	0.627
IL10	2.5 (2.1)	3.3 (2.7)	2.2 (1.8)	0.042
IL12p70	3.9 (2.3)	4.4 (2.0)	3.8 (2.4)	0.319
IL13	4.5 (7.9)	4.3 (4.7)	4.6 (8.8)	0.680
IL15	18.9 (18.5)	18.3 (12.0)	19.1 (20.6)	0.940
IL17a	12.9 (15.4)	13.3 (10.5)	12.7 (17.0)	0.823
IL18	61.9 (60.5)	64.6 (69.5)	60.9 (57.0)	0.455
IL1RA	1401.4 (3269.6)	2066.2 (4889.3)	1140.8 (2350.3)	0.146
IL2	18.3 (14.8)	19.3 (14.8)	17.9 (14.9)	0.551
IL21	219.0 (1819.0)	726.5 (3416.2)	20.1 (68.0)	0.111
IL22	1044.7 (9286.5)	3580.6 (17,458.7)	51.0 (175.8)	0.210
IL23	9.5 (9.6)	11.9 (10.8)	8.6 (8.9)	0.082
IL27	84.3 (210.0)	66.6 (98.5)	91.2 (240.3)	0.963
IL31	7.5 (9.8)	8.5 (7.3)	7.1 (10.7)	0.338
IL4	8.2 (8.6)	8.3 (6.0)	8.1 (9.4)	0.741
IL5	31.3 (91.2)	26.3 (43.0)	33.3 (104.4)	0.765
IL6	23.7 (40.7)	24.8 (24.4)	23.3 (45.7)	0.839
IL7	2.5 (2.5)	2.2 (1.8)	2.7 (2.7)	0.589
IL8	4.7 (9.2)	5.5 (13.9)	4.4 (6.7)	0.825
IL9	2.7 (2.2)	3.3 (2.2)	2.5 (2.1)	0.356
IP1b	91.3 (194.8)	147.9 (330.6)	69.2 (96.4)	0.116
IP10	60.9 (61.6)	56.6 (37.5)	62.6 (69.0)	0.877
LIF	20.2 (18.4)	19.6 (13.1)	20.4 (20.2)	0.935
MCP1	53.9 (71.8)	44.1 (35.6)	57.8 (81.7)	0.494
MIP1a	12.1 (28.2)	12.7 (23.9)	11.8 (29.9)	0.725
NGFb	4.6 (3.5)	4.6 (1.7)	4.6 (4.0)	0.873
PDGFBB	754.2 (1228.7)	763.1 (1352.3)	750.7 (1186.5)	0.778
PIGF1	536.1 (3583.6)	1621.8 (6694.7)	110.6 (317.9)	0.071
RANTES	21.2 (92.7)	38.7 (71.1)	43.5 (100.4)	0.849
SCF	8.7 (8.3)	7.1 (4.6)	9.3 (9.3)	0.676
SDF1a	35,867.8 (277,576.6)	95,765.4 (507,463.6)	12,394.4 (83,059.0)	0.093
TNFa	12.1 (15.5)	11.4 (10.6)	12.4 (17.1)	0.981
TNFb	3.8 (2.6)	4.6 (2.6)	3.5 (2.5)	0.278
VEGFA	604.6 (2735.0)	1394.5 (5059.8)	295.2 (535.9)	0.101
VEGFD	16.2 (17.3)	12.9 (8.2)	17.5 (19.7)	0.600

## Discussion

In the present study, we compared, for the first time, the cytokine profile between patients with and without headache during COVID-19 infection. In this pilot study, we analysed 45 different cytokines and interleukins. The main finding of our study was that IL-10 levels were significantly higher in patients with headache while other interleukins, such as IL-23 and PIGF1, also showed a trend to be higher in this group.

Our results should be interpreted with caution. In ideal conditions, considering that COVID-19 is a dynamic disease, the analytic parameters should have been obtained in the same stage of disease, and after the same time since the onset of the clinical symptoms. However, in order to make this study reproducible, the samples were collected in the first extraction after the hospital admission. We tried to minimize this problem by statistically adjusting for days of evolution of the symptoms, but this adjustment subtracted statistical power to the study. Besides, this was an exploratory study and instead of testing a single hypothesis, a high number of different cytokines were studied.

Despite the cytokine storm has been hypothesized as one of the possible mechanisms underlying headache in COVID-19 patients [13], few previous studies had analysed the relationship between cytokines levels and headache. To date, only IL-6 levels have been studied in COVID-19 patients with and without headache, in retrospective studies with contradictory results [6, 14]. Studies addressing differences in other interleukins were still lacking in the literature.

Our findings could support the hypothesis of a cytokine mediated mechanism underlying headache in COVID-19. The relationship between cytokines and headache has been studied for a long time [15]. The external administration of different cytokines, such as TNF, INF alfa, INF beta, INF gamma or IL-2, causes headache in humans [16, 17]. In addition, several studies have found relationship between primary headaches and elevated levels of cytokines. Regarding migraine, although there are discrepancies in literature, probably as a consequence of different patterns of sample collection relative to the time of attack [18], elevated serum levels of TNF a, IL-1b, IL-6, GM-CSF and IL-10 have been found during attacks and in attack free intervals [19–23]. Tension type headache also have been associated with elevated levels of IL-6 or IL-8 [24, 25]. Finally, in the case of systemic infections, cytokine cascade is thought to be the primary mechanism of headache and other accompanied symptoms, as fatigue, anorexia or nausea [11, 26].

Several studies have suggested a main role of the cytokine storm in the evolution of COVID-19 illness [9, 10, 27]. This supports the idea of this mechanism as the cause of headache, compared to other suggested hypotheses such as direct viral invasion of the central nervous system, hypoxia or dehydration [13]. In addition, the clinical similarities between COVID-19 and headache associated to viral systemic infections are in this line [7].

Pain secondary to cytokine is thought to be secondary to the activation of nociceptive sensory neurons and the nerve inflammation by proinflammatory cytokines inducing central sensitization [28]. IL-23 and PIGF1 are both pro-inflammatory cytokines [29, 30] so the finding of a trend to significant higher levels in our sample is in this line. On the other hand, in our study we found elevated levels of IL-10, an anti-inflammatory cytokine with a major role in mitigating inflammation through the ability to inhibit synthesis of non-specific proinflammatory cytokines such as IL-1, IL-6, TNF [31], In view of its anti-inflammatory properties, the IL-10 elevated levels in our sample may represent a homeostatic response to counteract the effects of other cytokines released during acute COVID-19, reflecting a more intense immunologic response to the virus in patients who develop headache. This hypothesis could explain the better outcome in COVID-19 patients with headache, compared with those patients without headache, observed in previous studies [4, 14]. The role of many interleukins and cytokines in COVID-19 infection is still unknown, some of them could be different depending on the presence of comorbidities, while others could be more related with the clinical presentation and severity of the disease. The role of a pre-existing headache in the cytokine profile also needs to be clarified.

Finally, if our findings are confirmed by further studies, they might have relevant implications. The attribution of a central role to the cytokine storm in COVID-19 pathophysiology led to different therapeutic approaches targeting the pro-inflammatory body state [32] or different cytokines [33, 34]. In the same way, knowing the physiopathology of COVID-19 headache and which inflammatory factors are implicated, could be helpful in the search of targeting therapies for persistent or treatment resistant headache in COVID-19 patients.

Another striking finding in our study was the association of headache and anosmia in patients with COVID-19. This finding was also described in other previous studies [35, 36]. The possible underling mechanism of anosmia still remains unknown on the one hand, clinical studies suggested that it could be related with the binding of the virus to the angiotensin converting enzyme type 2 receptors in the respiratory mucosae [37] which could explain the frontal topography of the headache. On the other hand, other authors suggest an inflammatory underlying mechanism of anosmia, and in this line a previous study found that a proinflammatory cytokine -TNF alpha- was higher in the olfactory mucosa in patients with COVID-19 [38].

Despite both osmophobia and anosmia might be related with the olfactory nerve, this symptom may be hidden due to the presence of anosmia. The frequency of anosmia in the general population before the pandemic was almost testimonial and mainly related with other viral infection or Parkinson disease [39], while osmophobia is one of the most specific symptoms of migraine [40]. Some authors suggest that COVID-19 might unravel a personal “migrainous biology” in some cases [41], but this is also still to be disentangled.

This study has important limitations. As previously mentioned, the analytic parameters were obtained in the same first day of hospitalization, but in different moments of the disease, which may reflect different inflammatory stages. On the other hand, the sample size was small, which limited the power of the study. The sample only included hospitalized patients, which could imply a selection bias. Finally, there are some statistical issues, as the large number of parameters that were compared which could increase false positive results. Despite these limitations, we consider that this pilot study may be helpful for the design of future validation studies.

## Conclusions

Selected cytokine levels are increased in COVID-19 patients with headache in comparison to COVID-19 patients without headache. The cross-sectional nature of our study impedes to draw any conclusions about causal relationship between cytokine levels and the headache. However, the higher levels of the anti-inflammatory cytokine IL10 found in our sample in patients with headache may be interpreted as a response to proinflammatory cytokine storm, reflecting a more efficient immune response in these patients.

## Supplementary Information

**Additional file 1.** Severity of COVID-19 disease according to the American Thoracic Society guidelines for community-acquired pneumonia.

## Data Availability

The datasets used and/or analysed during the current study are available from the corresponding author on reasonable request.

## Abbreviations

COVID-19: Coronavirus disease 2019
STARD: Standards for Reporting or Diagnostic Accuracy Studies
ICU: Intensive care unit
ELISA: Enzyme-linked immunosorbent assay
BDNF: Brain-derived neurotrophic factor
EGF: Epidermal growth factor
FGF-2: Fibroblast growth factor 2
GM-CSF: Granulocyte macrophage colony-stimulating factor
GRO: Growth-regulated oncogene
CXCL1: Chemokine (C-X-C motif) ligand 1
HGF: Hepatocyte growth factor
NGF: Nerve growth factor
LIF: Leukaemia inhibitory factor
IFN: Interferon
IL: Interleukin
IL-1RA: Interleukin 1 receptor antagonist
IP-10: Interferon gamma-induced protein 10
CXCL10: Hemokine (C-X-C motif) ligand 10
MCP-1: Monocyte chemoattractant protein 1
CCL2: Chemokine (C-C motif) ligand 2
MIP-1: Macrophage inflammatory protein 1
CCL3: Chemokine (C-C-motif) ligand 3
RANTES: Regulated upon activation normal T Cell expressed and presumably secreted
CCL5: Chemokine (C-C motif) ligand 5
SDF-1: Stromal-cell derived factor 1
TNF: Tumoral necrosis factor
LTA: Lymphotoxin alpha
PDGF: Platelet-derived growth factor
PIGF-1: Placental growth factor
SCF: Stem cell factor
VEGF: Vascular endothelial growth factor
SD: Standard deviation
IQR: Inter-quartile range
