# Supplementary material for: Cytokine and interleukin profile in patients with headache and COVID-19: A pilot, CASE-control, study on 104 patients
Source: J Headache Pain. 2021 Jun 4;22(1):51. doi: 10.1186/s10194-021-01268-w (PMC8177251; doi:10.1186/s10194-021-01268-w)
Supplement: Supplementary file 1 — Additional file 1. Severity of COVID-19 disease according to the American Thoracic Society guidelines for community-acquired pneumonia. [file 10194_2021_1268_MOESM1_ESM.docx]

Severity of COVID-19 disease according to the American Thoracic Society guidelines for community-acquired pneumonia.

| **Severity** | **Description** |
| --- | --- |
| Mild illness | Patients with uncomplicated upper respiratory tract viral infection symptoms and have non-specific symptoms such as fever, fatigue, cough (with or without sputum production), anorexia, malaise, muscle pain, sore throat, dyspnea, nasal congestion, diarrhea, nausea or vomiting. |
| Pneumonia | Presence of pneumonia but no signs of severe pneumonia and no need for supplemental oxygen.  CURB scale ≤ 1. |
| Severe pneumonia | Confirmed respiratory infection, plus one of the following:   1. Respiratory rate > 30 breaths/min 2. Severe respiratory distress 3. SpO_2_ ≤ 93% on room air |
| Acute respiratory distress syndrome | **Onset:** within 1 week of a known clinical insult or new or worsening respiratory symptoms.  **Chest imaging (radiograph, CT scan, or lung ultrasound):** bilateral opacities, not fully explained by volume overload, lobar or lung collapse, or nodules.  **Origin of pulmonary infiltrates:** respiratory failure not fully explained by cardiac failure or fluid overload. Need objective assessment (e.g. echocardiography) to exclude hydrostatic cause of infiltrates/oedema if no risk factor presents.  **Oxygenation impairment in adults:**   - Mild ARDS: 200 mmHg < PaO_2_/FiO_2_ ≤ 300 mmHg (with PEEP or CPAP ≥ 5 cmH_2_O, or non-ventilated) - Moderate ARDS: 100 mmHg < PaO_2_/FiO_2_ ≤ 200 mmHg (with PEEP ≥ 5 cmH_2_O, or non-ventilated) - Severe ARDS: PaO_2_/FiO_2_ ≤ 100 mmHg (with PEEP ≥ 5 cmH_2_O, or non-ventilated) - When PaO_2_ is not available, SpO_2_/FiO_2_ ≤ 315 implies ARDS (including in non-ventilated patients) |

*Sp: Saturation percentage. ADRS: Acute Distress Respiratory Syndrome. CT: Cranial Tomography. PaO_2_: Partial pressure of Oxygen. FiO_2_: Fraction of inspired Oxygen. PEEP: Positive end-expiratory pressure. CPAP: Continuous positive airway pressure.*

| IL1a | IL1b | IL1RA | IL2 |
| --- | --- | --- | --- |
| IL4 | IL5 | IL6 | IL7 |
| IL8 | IL9 | IL10 | IL12p70 |
| IL13 | IL15 | IL17a | IL18 |
| IL21 | IL22 | IL23 | IL27 |
| IL31 | IP1b | IP10 |  |

**Clinical variables that were analyzed:**We assessed the presence of general symptoms, including anosmia, arthralgia, asthenia, chest pain, cough, diarrhea, dyspnea, expectoration, fever, lightheadedness, myalgia, odynophagia, syncope, vomiting and weakness.
